# Women’s engagement with community perinatal mental health services: a realist evaluation

**DOI:** 10.1186/s12888-024-05804-1

**Published:** 2024-07-08

**Authors:** L. Fisher, A. Davey, G. Wong, S. Morgan-Trimmer, L. M. Howard, H. Sharp, K. H. Atmore, J. Brook, G. Collins, J. Domoney, E. Makinde, C. McCree, Heather A O’Mahen

**Affiliations:** 1 Department of Primary Care and Mental Health, Institute of Population Health, Faculty of Health and Life Sciences, University of Liverpool, Liverpool, UK; 2 Mood Disorders Centre, Psychology Department, Faculty of Health and Life Sciences, University of Exeter, Washington Singer Laboratories, Perry Road, Exeter, EX4 4QG UK; 3 Nuffield Department of Primary Care Health Sciences, University of Oxford, Oxford, UK; 4 Department of Health and Community Sciences, University of Exeter Medical School, Exeter, UK; 5 Section of Women’s Mental Health, Institute of Psychiatry, Psychology and Neuroscience, King’s College London, London, UK; 6 Department of Global Health and Social Medicine, School of Global Affairs, King’s College London, London, UK; 7 The Tavistock and Portman NHS Foundation Trust, London, UK; 8 Centre for Parent and Child Support and Community Perinatal services, South London and Maudsley NHS Foundation Trust, London, UK

**Keywords:** Perinatal Mental Health, Service Engagement, Patient experience, Realist evaluation, Qualitative

## Abstract

**Background:**

In recognition of the burden of Perinatal Mental Health problems, NHS England invested £365 million to transform women’s access to mental health care, including investment in Community Perinatal Mental Health Services. This study examined how elements of provider care affected women’s engagement with these services.

**Methods:**

Semi-structured interviews were conducted with 139 women and explored their experiences of care from 10 different Community Perinatal Mental Health Teams; including which service components participants believed made a difference to their initial and continued engagement. Realist analysis was used to create context-mechanism-outcome configurations (CMOCs) across interviews, since not all parts of the configurations were always articulated within singular interviews.

**Results:**

Four key pillars for engagement were identified: perinatal competence, relationship building, accurate reassurance, and reliability. The way perinatal competencies were relayed to women mattered; compassion, understanding and consistency were critical interactional styles. The extent to which these factors affected women’s engagement varied by their context and personal characteristics.

**Conclusions:**

As mental health problems increase, disproportionately affecting vulnerable populations, it is critical to continue to ensure support is not only available, but appropriately meets the needs of those individuals. Our findings suggest that key staff behaviours applied at the right time can support women’s engagement and potentially contribute to better treatment outcomes.

**Supplementary Information:**

The online version contains supplementary material available at 10.1186/s12888-024-05804-1.

## Introduction

### Rationale for evaluation

Perinatal mental health disorders are a serious public health issue, associated with poor outcomes for women (psychological and physical morbidity including suicide and life-threatening complications) [1], their foetus (preterm birth, low birth weight) and their infants/children [2–4]. In the UK, perinatal mental health (PMH) disorders are estimated to cost the economy £8.1 billion for each one-year cohort of births [5], with 72% of these costs relating to the longitudinal impacts on the social, emotional and cognitive development of children and adolescents.

There are effective psychiatric and psychological treatments for perinatal mental health problems [6] and recent research suggests that the children of women with postnatal depression will have fewer developmental problems at age two when their mothers receive psychological treatment and additional parenting support [7]. In total, the evidence suggests that effective services could reduce the costs of PMH disorders. However, in the UK, as in other areas of the world, few women with PMH disorders and their infants (8–30%) access mental health care during the perinatal period. These rates are in notable contrast to rates of women’s mental health treatment outside the perinatal period (50%) [8]. It is therefore important to understand factors that promote perinatal women’s initial and ongoing engagement with appropriate mental health care.

Recent meta-syntheses of qualitative data suggest that lower rates of mental health treatment receipt during the perinatal period may be due to the complex barriers mothers face when trying to access appropriate, acceptable care [9]. Key barriers include a lack of service provision that is relevant to perinatal problems affecting mental health, delivered in accessible and flexible ways that meet the challenging and quickly-changing needs of parents navigating pregnancy and caring for an infant [10].

### Programme initiative and environment surrounding evaluation

In England, in recognition of the costs and burden of PMH problems and their low service access rates, NHS England invested £365 million to transform women’s access to mental health care, including investment in Community Perinatal Mental Health Teams (CPMHTs). These secondary care mental health services provide treatment for women with moderate to severe and/or complex mental health disorders during pregnancy and up to 24 months postpartum. CPMHTs are multidisciplinary, comprising of psychiatrists, mental health nurses, pharmacists, psychologists, occupational therapists, nursery nurses and peer support workers. CPMHTs include; perinatal specialist assessment, psychiatric and psychological treatment, parent-infant interventions, and advice/liaison for primary care/maternity/secondary generic mental health services on identifying, preventing and caring for mild to severe PMH problems [11]. With the substantial growth in these services, which aim to see 66,000 women/year nationally, key questions arise about whether they are improving women and babies’ access to care, and what factors promote women’s successful initial engagement and ongoing adherence with mental health treatment.

Although previous literature has highlighted both generic and perinatal specific factors that promote engagement and adherence with mental health interventions [12–15], there has not yet been an evaluation of whether CPMHTs, which are designed to target multiple perinatal barriers and facilitators to accessing care at once, are successful in these aims. In the perinatal period, the experience of childbearing, changes in relationships, and exposure to scenarios in which a person may experience a lack of control mean this is a vulnerable time for women [16]. This may be especially true for those with histories of mental health difficulties, trauma, abuse and discrimination, experiences that are common amongst female mental health service users [17–20]. Early research into engagement with PMH care found that women reported improved willingness to engage with services when providers were relatable, had expert knowledge about the ways in which pregnancy and parenting in the postnatal period influence mental health, and provided women with the time to discuss their needs and the ability to choose between treatment options [21]. What is less clear, however, is *when* and *why* these elements might be important for particular populations and/or presenting problems, and *how* they might be modified to meet the specific needs of women in the perinatal period. These questions are critical to understanding which factors ensure CPMHTs successfully deliver care.

### Rationale for using realist evaluation and research question

In this paper, we report a realist evaluation that examined how elements of CPMHTs provider care affected women’s engagement with services. The data reported in this paper come from a larger mixed-methods study examining the effectiveness and cost effectiveness of community perinatal mental health services in England (ESMI-II). The ESMI-II study was funded to produce evidence about the effectiveness of CPMHTs in improving mother and infant outcomes. This included undertaking a realist evaluation to investigate how, why and for whom CPMHTs configurations and components are effective at improving acceptability and engagement with care and consequently improving outcomes. This paper extends the literature that outlines core generic provider elements supporting engagement with mental health treatment by asking, “what *perinatal specific* elements of service provider care make a difference to women’s engagement and adherence, *for whom* and *why?”*

## Methods

### Ethical approval

The study was approved by the Southwest - Central Bristol Research Ethics Committee (Reference: 19/SW/0218).

### Study design

The ESMI-III study consisted of 4 overarching work packages focused around different aspects of CPMHTs, from the characterisation of service variations seen within CPMHT, the reliability, validity and clinical feasibility of observational measures for assessing parent-infant interaction quality within CPMHTs and the exploration of linked health data in relation to access to secondary care mental health services. The final work package was the evaluation of the effectiveness and cost effectiveness of the CPMHTs through research interviews with 139 women, 55 sources of significant support and 80 health and social care practitioners, across 10 different CPMHTs. This is the work package and data which this paper is based upon, with focus only on the women’s interview data.

### Participants

A total of 10 CPMHTs were purposefully selected for variations in different components and configurations including: level of mother-infant and/or psychological interventions, collaborative care (e.g. integrated with universal and adjunctive services), quality of integration (e.g. communication, mental health co-located with maternity care), delivering perinatally tailored care, including care coordination vs. co-working with generic mental health teams, meeting women’s needs (flexible, remote/in home, de-stigmatising, family centred). A purposive sampling approach (with maximum variation in characteristics) was taken to identify up to 10 women who had received care in each service to help us refine and test aspects of our initial programme theory. Where possible, women were purposively sampled with different types of mental health problems (i.e., Emotionally Unstable Personality Disorder (EUPD), Anxiety Disorder, Trauma/Post Traumatic Stress Disorder (PTSD), Obsessive Compulsive Disorder (OCD), Severe Depression and Severe Mental Illness (SMI; Psychosis, Bipolar I) and across diverse sociodemographic groups. The current study focused on women’s experiences of CPMHTs and what they reported worked for them in relation to their engagement with the service.

### Recruitment

Recruitment of women took place between April 2020 and June 2021 and included those who experienced care prior to and during the COVID 19 pandemic. A member of the direct care team approached potential participants via phone or email and gave a brief overview of the study and an opt-out option. A link to more detailed information and the information sheet was then emailed to interested participants, including a consent to be contacted by the research team. Interested participants were then contacted by the researcher via telephone to discuss the study further. Participants were interviewed towards the end of their treatment or after they had completed their treatment, to avoid Hawthorne effects.

### Data collection

Semi-structured interviews lasting approximately 90 min, were conducted via telephone or videoconference with participants. Interviews followed a guide that was developed in collaboration with clinicians, policy makers and persons with lived experience. The interviews explored women’s experiences of care including their mental health history, access to service, and which service components participants believed made a difference to their access, engagement and adherence with treatment and the impact (if any) on their mental health/well-being, functioning and their relationship with their family, including their infant.

### Data analysis

Interviews were audio-recorded, transcribed verbatim and analysed using NVivo 12 software. Initially, interview data was coded using thematic analysis, focusing around broad areas of interest from the initial programme theory, including aspects such as referral process, access to care and quality of care. Further analysis involved the use of a realist logic of analysis with the goal of using the collected data to develop and refine the initial programme theory (see additional file 1) into a more refined realist programme theory. Data coding was deductive (informed by our initial programme theory), inductive (from the data within transcripts) and retroductive (where inferences were made based on interpretations of the data within sources about underlying causal processes – i.e. mechanisms). Through this approach, context-mechanism-outcome configurations (CMOCs) were developed and data to inform our interpretation of the relationships between contexts, mechanisms and outcomes was sought across interviews (e.g. mechanisms inferred from one interview could help explain the way contexts influenced outcomes in a different interview). Synthesising data from different interviews was often necessary to compile CMOCs, since not all parts of the configurations were always articulated in a single interview. We moved iteratively between the analysis of specific examples, refinement of programme theory, and any data we had collected to test particular theories.

## Results

### Details of participants

Characteristics of the women are described in Table 1.

**Table 1:** Participant demographics

**Age (Mean 32.2,** ***SD*** **5.20, range 20–45) (** ***N*** ** = 139)**	
≤ 25	*12* (8.63%)
26–30	*38* (27.34%)
31–35	*53* (38.13%)
36–40	*26* (18.71%)
41–45	*10* (7.19%)
**Ethnicity (** ***N*** ** = 139)**	
White	*110* (79.14%)
Black/African/Caribbean/Black British	*10* (7.19%)
Asian/Asian British	9 (6.47%)
Mixed/Multiple ethnic groups	6 (4.32%)
Other ethnic group	4 (2.88%)
**Primary Diagnosis (** ***N*** ** = 136)**	
Severe Mental Illness	*23* (16.9%)
Personality Disorder	*16* (11.8%)
PTSD and OCD	*22* (16.2%)
Depression and Anxiety	*75* (55.1%)
**Employment Status (** ***N*** ** = 137)**	
Employed	69 (50.36%)
Unemployed	30 (21.9%)
Homemaker/Stay at home Mum	14 (10.22%)
In education	1 (0.73%)
Long term sick	5 (3.65%)
Maternity leave	18 (13.14%)
**Relationship Status (** ***N*** ** = 138)**	
Married	74 (53.62%)
Cohabiting	38 (27.54%)
Living separately	5 (3.62%)
Divorced	2 (1.45%)
Single	19 (13.77%)
**Socioeconomic deprivation (** ***N*** **= 135)** Quintile 1 = most deprived; Quintile 5 = least deprived	
1	17 (12.59%)
2	29 (21.48%)
3	30 (22.22%)
4	34 (25.19%)
5	25 (18.52%)

### Main findings

**Fig. 1:**
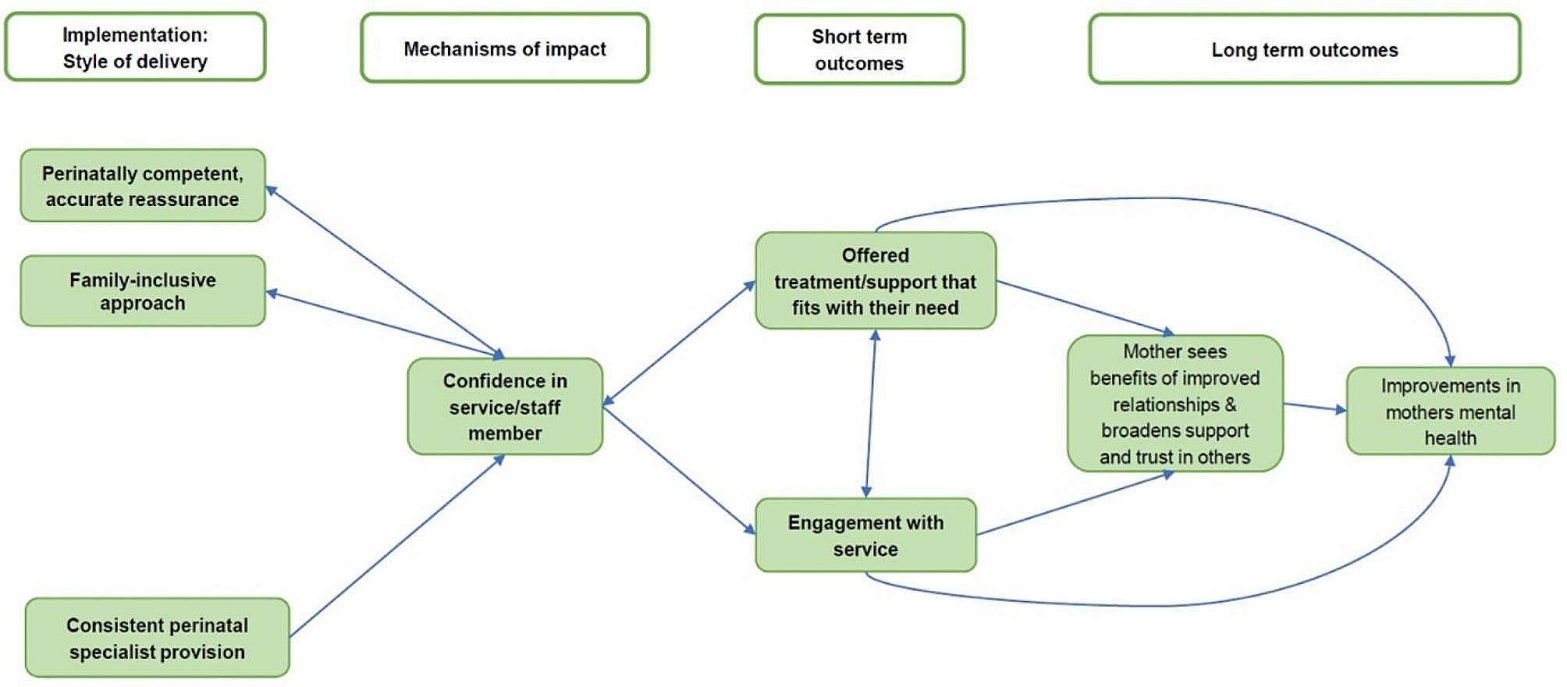
Overall programme theory of women’s engagement with CPMHTs

An initial programme theory was designed prior to data collection to explain and account for engagement and its association with perceived outcomes. Based on collected data this was gradually refined into Fig. 1. In this figure, using the hypothesised elements from the initial programme theory, we mapped high level patterns observed within the data that were associated with women’s engagement with the service.

**Fig. 2:**
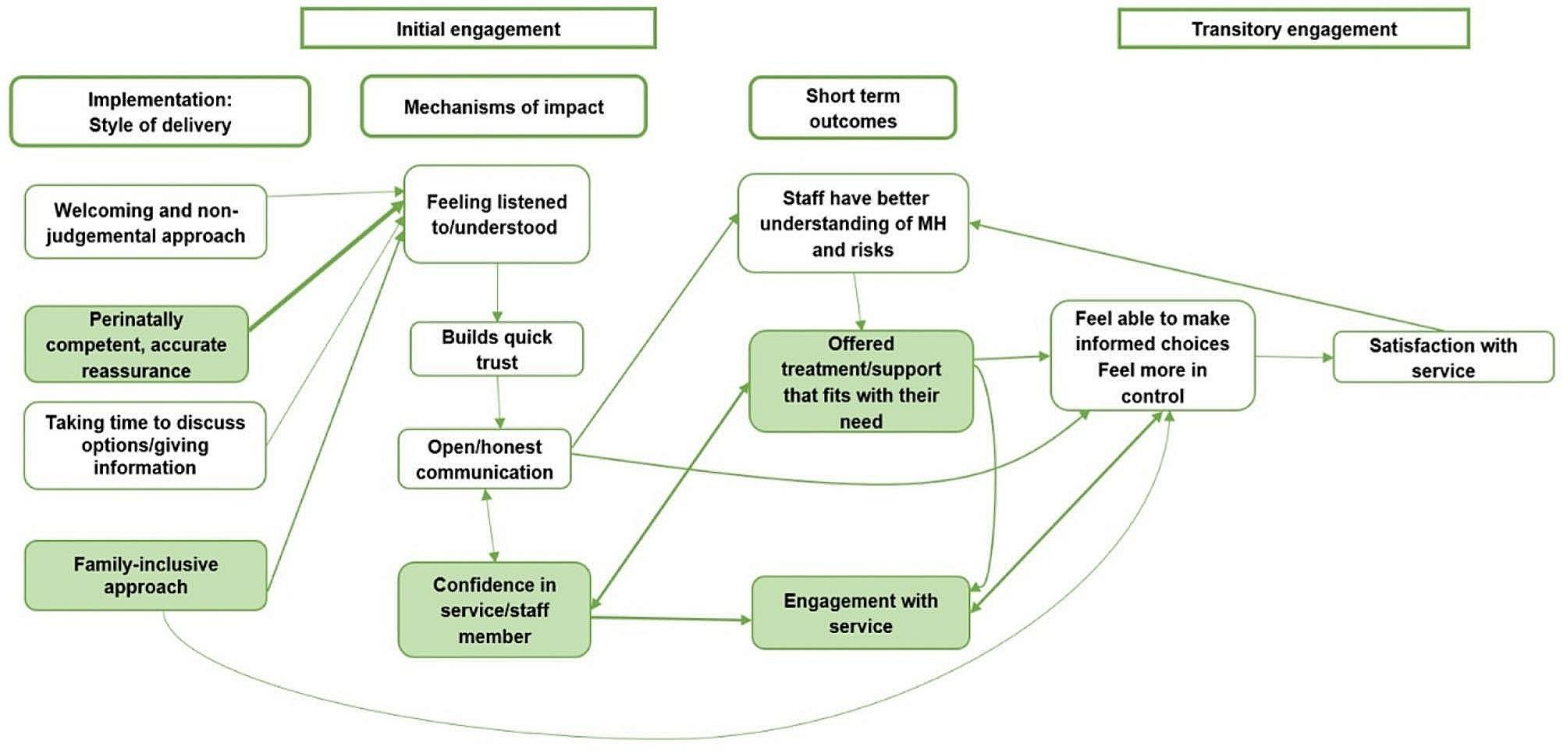
Programme Theory specific to the initial engagement phase

**Fig. 3:**
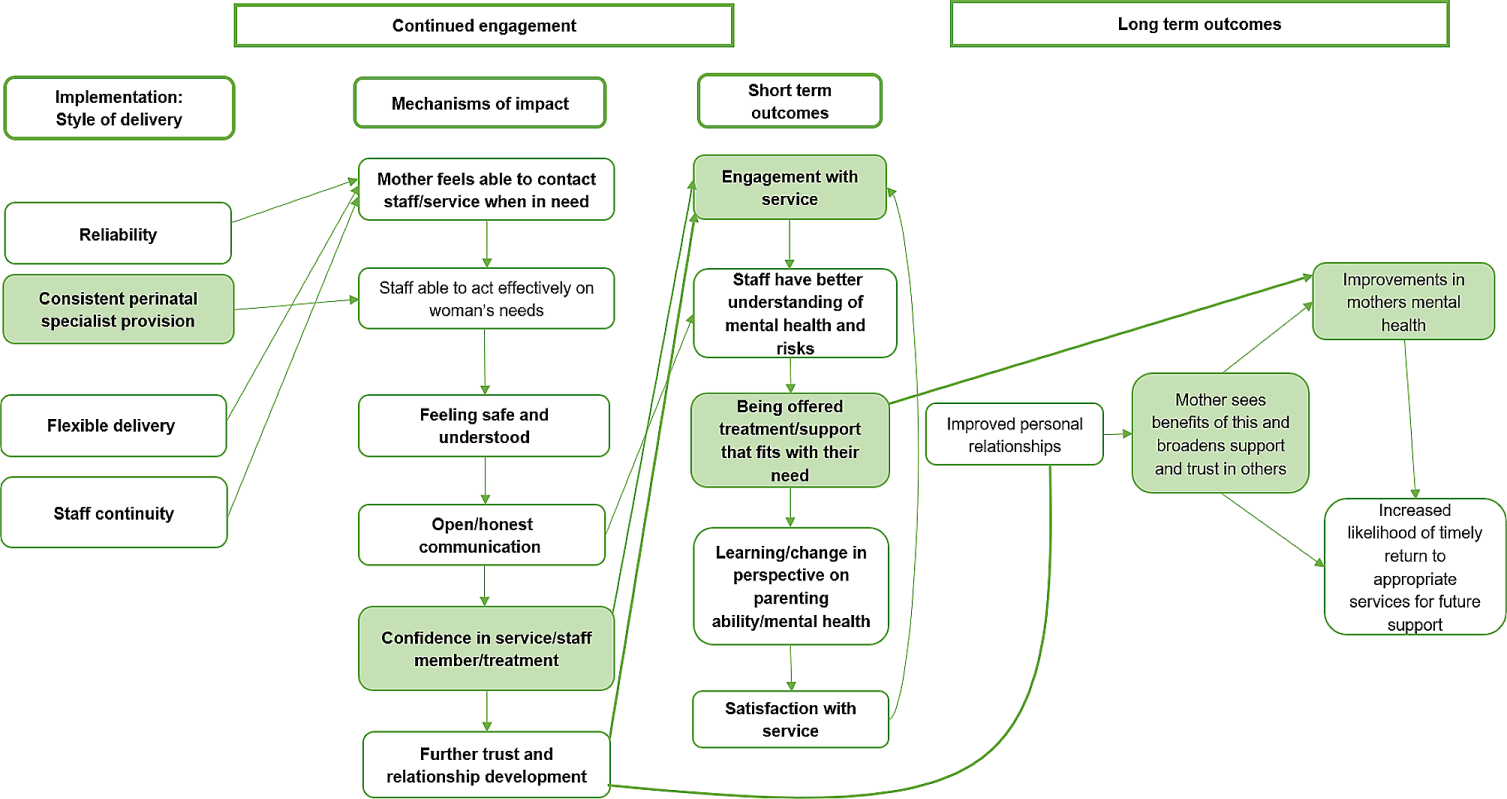
Programme Theory specific to continued engagement with CPMHTs

Figures 2 and 3 explain the engagement processes in more detail. We report the processes and effects of engagement in three stages: Fig. 2 illustrates initial engagement, which we defined as the process of referral, the first contact, the woman’s assessment appointment with the service and the first contact with their respective key worker(s). We also included transitory engagement in Fig. 2, which we defined as the process between the initial assessment appointment and when the woman agrees an initial treatment plan with the service. Figure 3 (presented later in the findings) illustrates continued engagement/adherence, which we defined as the woman’s ongoing subsequent treatment contacts with the service. In Figs. 2 and 3 we portray both the direct primary relationships between these high-level factors, and the indirect factors supporting these relationships as they relate to engagement. The boxes highlighted in green are components which appear within and are taken from Fig. 1 and the unfilled boxes are additional details not previously covered within the overall programme theory. Within the following findings, we present knowledge claims supported by CMO configurations numbered 1–19 [e.g., CMO-C1]. See Tables 2, 3, 4, 5, 6, 7 and 8.

### Initial engagement- providing reassurance

Before accessing CPMHTs, most women described feeling vulnerable, confused about what kind of support might be helpful, and frustrated with the help-seeking process. They reported struggling with significant concerns about stigma and feeling they were failures as mothers. A significant proportion of women in the study stated they were afraid that having mental health difficulties put them at risk of losing their children. In that context, mothers reported that their fears of judgement by providers were a key barrier to engagement with mental health services.

Fear of judgement was especially acute in mothers who had had contact with generic mental health services prior to accessing the CPMHTs. These women reported they were particularly wary of mental health services because they had encountered poor understanding of their perinatal specific needs, judgement, and a lack of perinatally-tailored interventions in generic mental health services. For example, women reported receiving incorrect guidance about medication usage from GPs or psychiatrists in generic adult community mental health teams. They also reported that the psychological interventions they received from generic services lacked a focus on perinatal specific mental health difficulties.

*“[Adult Mental Health] took me off of my medication…and put me on a different one that they classed as safer to be on when pregnant. But since being in perinatal, the doctor there said that they should not have done that…I could have stayed on the medication that I was on throughout the pregnancy and it would have actually [been] safer”* [2M5, EUPD, OCD, Anxiety & Depression].

*“I went to [primary care mental health, Improving Access to Psychological Therapy; IAPT] groups, like a panic group…just trying to help with like different strategies and stuff like that but it didn’t really apply to me because I was pregnant, a lot of my worries, panics, fears were about were around pregnancy…it was really hard for me to actually engage with the service because it wasn’t for pregnancy, it was just for people who weren’t pregnant.”* [6-M2, Anxiety & Depression].

Some women consequently approached CPMHTs apprehensively but were positively and powerfully impacted by initial conversations with CPMHTs staff who took the time to hear their worries and problems, were perceived as knowledgeable in addressing their perinatal specific needs and normalised the purpose of the service. [CMO-C1] [CMO-C2] are presented in Table 2.

*“They gave me so much like advice…like other mums go through this…they kind of went through stories like confidentially… ‘this mum does this, or this mum was with us for this long time and here they are now’ and that kind of made it better”* [9-M6, Depression and bonding difficulties].

**Table 2:** Initial engagement and providing reassurance CMOCs

**CMO-C1**		
**Context** When CPMHTs staff take the time to understand and respond to women who have had previous negative experiences with services	**Mechanism** They feel they are in safe hands	**Outcome** Women are more likely to engage (and continue to engage with the service)
**CMO-C2**		
**Context** When staff members give time within appointments to identify and respond appropriately to women’s concerns and expectations	**Mechanism** They feel listened to and understood	**Outcome** • They are less anxious and more open to being under the service • They have an initial confidence/trust in the staff/service

Relieved that they were in the right place and with the right providers, many women expressed gratitude when they perceived they had *accurate reassurance* that they were doing the right thing for their mental health and their baby by accessing the service. Critically, women said that a key factor supporting their engagement was when staff addressed their most significant fears - that they were not a terrible mother or person, that their baby wouldn’t be taken away (when this was the case) - and when staff highlighted what women were doing well. [CMO-C3] as shown in Table 3.

*“I think my problem is I’m a lot more capable than I think I am and the anxiety just completely muddles my brain and what [perinatal nursery nurse] and [perinatal mental health nurse] did was allow me to access the tools I already have, you know, I am a really good mum, I am switched on, you know, I had already done a lot of the prep for welcoming a new baby and [perinatal nursery nurse] just, you know, gently guided, reassured me that, you know, ‘hey, look at everything you’re doing’.”* [5-M10, Anxiety & Depression].

Women’s rapidly growing confidence in both the expert perinatal competence of staff and their ability to address problems non-judgementally allowed them to engage openly with staff if they struggled with challenging issues. [CMO-4] [CMO-5] as presented in Table 3. For example, women who had safeguarding issues reported they appreciated when staff openly and honestly acknowledged that social care might need to become involved, explaining how the CPMHTs and social care would be there to act in the mother and baby’s best interests.

*“you are slightly guarded about how mental you tell someone you are [laughs], because you do worry about, you know, will they take my children away from me, you know, what intervention is going to happen next? but … I’m not in that position where I ever felt that, ‘oh, I can’t say that’… [perinatal nursery nurse] was incredibly helpful and supportive through that process”* [1-M8, Depression].

“[scan practitioner] said to me, you know, ‘I’m going to put a yellow flag up there for you, just because of your mental health’…she made me almost feel like I needed social services involved because I was mentally not well, whereas, when [perinatal mental health nurse] come round, she explained to me that if social services were to be involved, it would be for the benefit of me, not to try and rip my child away from me.” [7-M10, Anxiety & Depression].

**Table 3:** Initial engagement and providing reassurance CMOCs – confidence

**CMO-C3**		
**Context** When staff members provide accurate and knowledgeable reassurance to women who have safeguarding concerns about being under the service	**Mechanism** They do not feel they are being unjustifiably judged	**Outcome** They start to build trust and confidence in the service
**CMO-C4**		
**Context** When women feel they are not judged, supported and less anxious about their engagement	**Mechanism** They feel they can have confidence that the service has their best interest at heart	**Outcome** They begin to build honest relationships with staff members
**CMO-C5**		
**Context** When women have confidence that staff are there to act in their best interest	**Mechanism** They feel safe to share information	**Outcome** They are more likely to disclose sensitive information relevant to their care

In sum, perceived staff expertise and perinatal competence [CMO-C6] (see Table 4), combined with their non-judgemental reassurance, gave many women a sense of feeling understood and safety, which provided them with a foundation of trust and confidence in the service. [CMO-2] as presented in Table 2 previously. Though still wary and vulnerable, women described this initial confidence as key in their willingness to “take a leap of faith” and engage openly with the CPMHTs. Some of these women reported they were surprised at how open they were able to be with perinatal staff, because of the way staff modelled honest and non-judgmental communication. [CMO-C7][CMO-C8] as presented in Table 4.

*“[perinatal psychologist] said to me ‘you know I am not here to judge you, because other people think the same as you’ … after that conversation I never once thought ‘I can’t possibly tell anyone that because they’re going to judge me.’ After that conversation I was completely honest with [perinatal mental health nurse] and [perinatal psychologist] which I never thought … I would be.”* [8-M13, Bipolar & Psychosis, Postnatal Depression & Anxiety].

Women’s ability to be open allowed staff to better understand the causes and contexts in which their mental health problems were occurring and how they were affecting women and their families, and increased the likelihood staff would correctly identify which treatments would be beneficial for their needs. [CMO-C9], as presented in Table 4.

*“I think it’s definitely someone listening to what the problem is and thinking about what might help you the most. And then with the acceptance and commitment therapy, yeah, that was the same, actually, thinking about it, [perinatal practitioner] really listening to me, hearing what I needed, where I was coming from… she had actually given me all the tools that I needed… I have felt completely supported, I know that I’ve always had someone to turn to, they’ve always been fighting my corner if I need anything, they’re amazing”* [1-M13, Bipolar II].

**Table 4:** Further initial engagement and providing reassurance CMOCs – honesty and openness

**CMO-C6**		
**Context** When staff show their specialist knowledge through discussion of women’s difficulties, needs and treatment options available	**Mechanism** They believe they are the right people to help them	**Outcome** Women feel confident in the service and staff
**CMO-C7**		
**Context** When women feel staff are being honest and open with them	**Mechanism** They have confidence in them	**Outcome** • Women are more likely to open up • Improves women’s engagement
**CMO-C8**		
**Context** When care provided by the staff and the service is perceived to be consistent and collaborative (e.g. not being told or forced in what to do, feeling they have a choice/say)	**Mechanism** They feel staff respect and value their input	**Outcome** Women are more likely to engage and be open and honest
**CMO-C9**		
**Context** When women feel able to be open about their needs and disclose information relevant to their care	**Mechanism** Staff are able to accurately assess women’s needs and give them the right support	**Outcome** Women are more likely to get appropriate treatments

***Transitory Engagement***.

In a cycle of growing trust, women stated that receiving treatment offers that matched their needs helped them to feel as if they had a greater degree of choice and control in their treatment, by virtue of being heard and recognised. [CMO-C10] as shown in Table 5.

*“That would always be a question in every consultation ‘well what do you think we can do to help you… Make you feel a bit better?’ …And sometimes there wasn’t anything and sometimes perhaps I’d bring something up that I think they could help me with and they’d say, ‘well we’ve got this person that might be able to help you’ and then obviously that was my choice.”* [9-M9, Postpartum Psychosis].

In turn, they were grateful for staff members perceived as competent in perinatal mental health, who were able to confidently deliver on treatment planning. This produced a sense of trusted security in the service, prompting greater readiness for continued engagement with the service. [CMO-C11] see Table 5.

*“I thought they were really good…experienced and understanding people…having people that are specifically…this is what they do for this specific situation…I felt like, you know, if there was something going on, then they’d be able to spot it a mile away, so I felt quite…reassured by that.”* [8-M17, EUPD, Psychosis].

**Table 5:** Transitory Engagement CMOCs

**CMO-C10**		
**Context** When staff consistently: • Give clear timely information and; • Listen to and flexibly accommodate women	**Mechanism** They feel understood by the specialist service and that they are being given the means to make an informed choice	**Outcome** They gain confidence in the service
**CMO-C11**		
**Context** When women continue to have confidence in the ability of the CPMHTs to meet their needs	**Mechanism** They believe and are hopeful that they can get better	**Outcome** They are both more satisfied and engaged with the service

In contrast, some women reported that they had less positive early interactions with staff. When staff failed to take the time to have open, non-judgmental discussions, women reported they felt coerced to follow treatment plans. This had a negative impact on their immediate mental health and resulted in reticence and anxiety about future contact. Many of these women, faced with few other appropriate treatment options, continued to guardedly engage with the service, though this was marked by avoidance. Women had poorer attendance at appointments or attended appointments alone, kept appointments brief and interacted no more than they felt they needed to. As a consequence, women failed to develop close and trusting relationships and services had to work harder to keep them engaged.

*“I didn’t see talking to her as a solution. It was more of a problem, because my first contact with her was about like trying to get me on this other medication. So, I’d got anxieties over talking to her again, because that’s how I’d kind of remembered her, from then on. So, I never really wanted to hang around with her, but I knew that if I needed help, I’d get it.”* [3-M7, Anxiety & Depression].

### Family inclusive approach

Women reported that when the perinatal team considered their needs within a family context this supported their engagement. Women noted they benefitted from getting help with parenting support in the context of struggling with their own mental health problems. Women also commented on how including a loved one in their care (if they wanted it) supported them to attend appointments, and aided and encouraged them to be honest and open about their experiences. [CMO-C12] presented in Table 6.

*“Sometimes, when I was struggling, it felt a bit hard to be open with people…So, having [Partner] there as support he kind of, you know, encourages me to be honest. And, because I’ve been honest with [Partner] about how I feel, he knows if I am struggling to say something, or explain something, [Partner] will like help to explain it, or…will, you know, reiterate what I’ve told him…So, it was helpful to have him there at times.”* [3-M10, EUPD, PTSD].

Family members also acted as valuable co-historians, and supported women in their care outside of the service. Critically, significant others could be an additional source of information and communication when the woman might be critically unwell and unable to make contact and engage with the service by themselves.

*“I felt a bit better after the meeting, knowing there were people, sort of in the background, I could contact if there was anything, or… my husband would contact them, ‘cos I wasn’t in a place where I would speak to people or ring them up”* [EX-BM2, Severe Depression].

This specialist ability to work closely with their loved ones and in a family context, helped women to feel that staff had their family’s best interests at heart and further reduced their feelings of shame about experiencing mental health difficulties as a parent.

[Interviewer: what’s important? “being sort of family-focused, or patient focused in the needs of the mother and the child and the family, I suppose. You know like how they’ve extended things for me, because of my situation…That’s been a massive one for me. Advocating and sort of just helping build confidence…empowering me really.” *[3-M11 EUPD, Postnatal Depression with Psychosis]*.

Some women, however, stated that they preferred not to have loved ones included in their treatment; these were often women who were experiencing bonding difficulties or suicidal ideation. Fearful of judgment and ashamed of their feelings, women described the appeal of seeking support from the CPMHTs as being something that was separate and confidential from their day-to-day lives. In being giving the choice to attend appointments alone, they felt more comfortable to be open about their experiences and needs. [CMO-C13] see Table 6.

*“I wouldn’t necessarily have wanted him in my appointments because I think that a lot of the things that I was feeling and saying at the time were really hurtful to (Husband). So, I don’t think that would have been helpful for either of us. I would have felt, you know, judged by him for saying things which of course he would be and he would have felt very uncomfortable about the way I was talking about our baby.”* [3-M6, Anxiety & Depression].

**Table 6:** Family inclusive approach CMOCs

**CMO-C12**		
**Context** When women wanted and opted to have loved ones at their appointments	**Mechanism** Those loves ones served as an additional support in helping the woman to voice her experiences or concerns	**Outcome** They were more likely to engage and be more honest with practitioners about their needs
**CMO-C13**		
**Context** When women do not want their loved ones to know the full extent of their difficulties and cause concern (e.g. experiencing bonding difficulties or suicidal ideation)	**Mechanism** They found their difficulties shameful and feared being judged	**Outcome** They most often chose not to include loves ones within appointments and felt better able to speak openly with practitioners

### Continuing engagement

As women continued in their treatment with the service, their focus shifted from factors that supported their initial engagement to factors that helped sustain them in a mental health journey that was for many a challenging, non-linear path, complicated by the challenges of adapting to a changing pregnancy and/or rapidly developing infant development across the first postnatal year. Against this background, women highlighted how service reliability and consistency, flexibility, and having a key identified member of staff in the team were critical to keeping them engaged with the service and their treatment.

### Reliability and consistency

Women reported that the maintenance of trust in the service was dependent on the reliability of staff, who needed to be consistently compassionate, non-judgemental, and deliver consistent messages both across time and between providers in the service (e.g. about medication use, treatment approaches). [CMO-C14] presented in Table 7. This reliability was particularly valued when women faced challenges in their own relationships that left them feeling vulnerable and distrustful. Reliability and the team’s shared, perinatal knowledge helped increase women’s confidence in the competence of staff and continued to reassure them that they were getting the right help for their difficulties. In turn, they noted that this contributed positively to their ongoing engagement with the service. [CMO-C15] see Table 7.

*“you didn’t lose your trust, you know, she made sure that the communication lines were open… that she followed through with what she was going to say and what she was going to do…you knew what to expect, there weren’t any surprises”* [5-M12, Psychosis].

Frequent contact also helped with engagement and was perceived to be a very different approach to how other services operate. Women who felt they had little or no external support, or who felt unable to discuss their difficulties with support networks, especially appreciated regular check-ins from staff, which they interpreted as a sign that staff cared about them, their needs and progress.

*“I think definitely with the phone calls it was definitely like a massive thing…It’s the frequency…it’s not like someone’s just coming in seeing you once a month, like you’re having [weekly contact], you’re building that relationship up with somebody and the only way that you can get, I think, very into a person’s mind is by having regular contact. They open up to your more and you find it more [a] trusting … relationship otherwise you don’t build it, … then people aren’t honest.”* [LV-NWM4, EUPD, Depression].

However, instances in which staff were not consistent or reliable, cancelling appointments or not showing up to appointments on time or at all, women felt frustrated and this eroded their confidence with the service or practitioner. Others at times felt ignored by practitioners. Women described some staff as being ‘dismissive,’ and ‘not passing along’ pertinent information about their care to other practitioners and noted how this negatively impacted their engagement with the service. [CMO-C16] see Table 7.

*“whenever I did seek help it was brushed off. So, in the end I did start to learn to like keep my mouth closed a lot of the time and not tell [perinatal practitioner] that I was struggling or anything.”* [3-M2, Anxiety & Depression, PTSD].

**Table 7:** Reliability and consistency CMOCs

**CMO-C14**		
**Context** When staff work collaboratively with each other to minimise mixed messages	**Mechanism** Women have confidence in the perinatal service	**Outcome** Reduces women’s anxieties
**CMO-C15**		
**Context** When practitioners repeatedly and consistently give time within appointments to identify and respond appropriately to women’s concerns and expectations	**Mechanism** They continue to feel listened to and understood	**Outcome** They continue to be less anxious and more open to being under the service and further develop their confidence/trust in the staff/service
**CMO-C16**		
When staff members are unreliable in their interactions with women	Women lose trust and confidence in the service	Impacts negatively on women’s engagement

### Key staff connection

For these reasons, women found having at least one person in the service whom they felt was their main worker helped them to feel safe, connected to the service, understood, and accurately represented to the rest of the service. Women who experienced a change of main practitioner during their care reflected on the negative impact this had on their recovery as they had to shift focus on their treatment to build trust and relationship with a new person. Some women who experienced multiple changes in staff described having lower and more guarded levels of engagement as a result. [CMO-C17] presented in Table 8. This was at times detrimental to a woman’s care, as a few described being less likely to contact the service even when their own safety and wellbeing was at risk.

*“I was just kind of feeling comfortable opening up to one person and then to be handed to somebody else and then again to somebody else who I never actually met. So, that kind of trust was never really built and …that shift has hindered me somewhat… I felt like I couldn’t really call anybody if I needed to.”* [6-M7, Postpartum Depression].

Some services adopted a different model, where women were assigned a small core group of practitioners involved in their care. Women in this service model reflected that they were less affected if they experienced changes in practitioners because they had good existing relationships with other practitioners in their core group. [CMO-C17] see Table 8.

*“I do think it’s helped that every time [perinatal mental health nurse]’s been off it’s always been [Occupational Therapist]. I think you feel a bit more supported than somebody just turning up that you don’t know.”* [9-M11, Bipolar I].

### Flexible delivery

While women valued consistency and reliability, many also reported that their ongoing ability to engage in the service required a flexible delivery approach. Women, faced in pregnancy with managing fatigue, feeling physically unwell, and multiple medical appointments, and postnatally with changeable infant care schedules and rapid developmental changes, found that the rigidity of generic mental health services clashed with their ever changing and unpredictable parenting demands and therefore hampered their engagement. In contrast, women described perinatal services as providing a more flexible, perinatally-informed approach that supported their ability to remain engaged with treatment. [CMO-C18] see Table 8. For example, services provided women with the means to contact individual staff members, and ensured appointment locations took place in environments in which the mother felt comfortable. Women reported this approach helped them to feel as if the team was exerting a real effort to meet their needs and to give them reasonable opportunities to receive the support they needed.

*“I was supposed to attend a course, which was for people just like myself, going through the perinatal situation, but I couldn’t make it and I was heartbroken. [Occupational Therapist] didn’t judge me…she did it with me, like, remotely… I was so much better than when I started. I actually felt it, as well, I felt supported, I felt sort of, like, ready to put my foot forward” [10-M1, Postnatal Depression]*.

### Impacts of engagement

Women described that they benefitted from services in two ways. Firstly, when they had honest relationships with staff and were able to get the right treatment [CMO-C9] (as presented in Table 4 previously), and secondly, when they formed trusting relationships with staff that they were then able to use as models to help them build trust with others in their support system and thereby indirectly get appropriate help in their day-to-day lives. [CMO-C19] as presented in Table 8. Strikingly, women reported that getting the right treatment and feeling able to engage in that treatment not only helped their wellbeing and functioning, but in some cases, saved their lives.

*“if I hadn’t had the perinatal mental health team, I think I would have probably been dead, or (baby) would have been dead to be honest with you. I was that ill.”* [2-M11, EUPD, Postnatal Depression with Psychosis].

*“the … therapeutic process meant that I began to understand how unboundaried I was and how that wasn’t really my fault and that actually, even though it felt really, really difficult, if I hadn’t gone through this process with them then my relationships with my family would probably be worse…it completely changed the relationship that I have with my mum, which I can just about handle now.”* [5-M3, Anxiety & Depression].

Further, drawing on their faith in perinatal mental health services, women reported that in the future they would be more likely to seek support earlier on when they needed it, critically supporting ongoing mental health.

*“I used to be very guarded about my mental health. So, I would not openly say to someone, ‘I’m a mental health patient’. No way. If I had another child I absolutely would and that kind of confidence and ability to admit that is down to the perinatal mental health intervention that I had. I’m not guarded about it anymore.”* [6-M1, Bipolar I].

**Table 8:** Key staff connection, flexible delivery and impacts of engagement CMOCs

**CMO-C17**		
**Context** When women were able to see the same familiar practitioners regularly	**Mechanism** • They felt the support was more efficient • They had built up trusting relationships	**Outcome** • They were happier with their engagement • They were more likely to seek support and keep honest and open in their communication
**CMO-C18**		
**Context** When professionals are willing to take account of women’s changeable and unpredictable infant care demands when providing services	**Mechanism** They value and need the flexibility	**Outcome** Women are more likely to continue to engage
**CMO-C19**		
**Context** When services help women to understand the value of opening up and being honest with others	**Mechanism** They see the benefit of doing so	**Outcome** They are more willing to be open with their loved ones

## Discussion

### Summary of findings

This study demonstrated four key pillars in the foundations of women’s engagement with perinatal mental health services. It demonstrated that women’s engagement was underpinned by their perceptions of service providers’ perinatal competence. The way perinatal competencies were relayed to women also mattered; compassion, accurate understanding and reliability and consistency were all critical interactional styles that helped build strong therapeutic relationships. The extent to which these factors affected women’s engagement varied by their context and personal characteristics.

### Comparison with existing literature and recommendations

We found that in perinatal mental health, first impressions count. Women were initially cautious about engaging with CPMHTs, though we found their reasons for wariness varied by personal characteristics. Stigma and fear of having one’s child removed from their care, common barriers to perinatal mental health service engagement [22], were significant for many women. However, these fears were compounded in two groups of women: those who did not have significant previous contact with mental health services, and those who had negative previous experiences with mental health services. The first group’s lack of knowledge of CPMHTs magnified their fears of stigmatization in what they identified as an already vulnerable period of life. The second group described many of their previous experiences of mental health care in negative terms and were guarded and sceptical of CPMHTs as a result. First contacts with the CPMHTs were therefore a key turning point for many women. What staff did in those initial contacts mattered.

Consistent with previous literature on therapeutic behaviours [13], a welcoming and non-judgemental approach was critical to women’s engagement both initially and beyond, but we found that this stance needed to be underpinned *by a firm base of perinatal knowledge and expertise*. With these elements in place, women reported they experienced staff as balanced, reflective and non-reactive. This was in contrast to the judgement and unpleasant experiences women reported they feared or had experienced from clinicians in generic mental health and health services. Non-judgemental and perinatally competent approaches helped women build *confidence and trust in staff* and reassured them the CPMHTs could help them. These key components promoted women’s hope for improvement and their willingness to engage *openly and honestly* with the service, mechanisms consistently highlighted across the therapy literature as essential for positive outcomes [22, 23].

Previous literature has noted the importance of reassurance in building patient engagement [24]. We found, however, that the quality and *accuracy* of reassurance mattered. Though accurate reassurance might include negative information (e.g., hospitalisation, social services involvement), women who trusted staff who were perinatally competent and had their best interests in mind reported they appreciated this honesty. It reduced uncertainty about their circumstances and gave them clarity about what steps they needed to take towards improvement.

There is increasing recognition of the importance of continuity of care in both health [25, 26] and mental health [27], with maternity services in many countries adopting small team, “case loading” approaches [28, 29]. Women in this study also described how having a continuous provider, or a core set of providers, was critical to their engagement. It ensured ongoing trust in the CPMHTs, patient openness and a sense that they had the agency to undertake the right set of treatments for them. Women in this study also noted that it was critical for staff to be flexible in how they delivered treatment and to undertake outreach, because of the uncertainties and pressures associated with pregnancy and caring for an infant [17]. These behaviours not only helped women to remain engaged in their treatment, but also provided them with stable interpersonal models. Consistent with the literature on transference in psychotherapy [30], some women reported that these interpersonal models also helped them to rebalance their own relationships and this supported their ongoing recovery.

Not all women interviewed had these experiences, however, and when elements in this unfolding set of staff behaviours were missing, women were less likely to actively engage with services [23, 31]. Notably, a number of women reported they still remained with the service, but had only minimal and guarded interactions with it.

Together, this study provides clarity on what CPMHTs can do to effectively engage perinatal women as shown in Table 9.

**Table 9:** Recommendations for services

What can staff do…	…and how will this increase engagement
1. Use perinatal specific knowledge to provide accurate reassurance to women	This not only normalises their experiences but can be very validating. With this being perinatally specific, women feel they can really trust in the advice and reassurance being given, which makes a huge impact when it comes to women engaging with the service and the advice given
2. Use your communication skills to build and sustain a therapeutic relationship with women	Women feel vulnerable and have multiple concerns about many things (e.g. their ability as a mother; or that their baby might be removed) when attending services. They need to feel that you have their best interest at heart before they will start to engage and open up
3. Be consistent in the messages you give to women about their experiences, what options are available and why these may be helpful	Consistency helps women to feel more confident and in instances where women may not be ready or open to an aspect of care initially e.g., a specific treatment; consistent messages around this can help women keep this in mind and enable them to consider options later in their care journey that may be beneficial. Consistency amongst messages given by individual members of staff is also important for increasing confidence in the service
4. If you do not know the answer to a query or concern that women have, tell them you will endeavour to find out from a professional whom would know the answer	This does not decrease confidence, but only serves to increase confidence and trust from the woman’s perspective in that they are getting the best service for their needs and that there are different professionals whom can be called upon when needed
5. Be flexible and use knowledge of the perinatal period and the individual you are seeing in order to balance timings of appointments, amount of outreach needed and the content of sessions	This makes it easier for the women to engage, when they feel the service and practitioner is being responsive to their needs and understands how these might change over time. Once they perceive that staff are responsive, they are more likely to get in touch when they need to rather than disengage
6. Talk honestly and openly with women about their experiences. Don’t shy away from difficult conversations	When women feel staff are being open with them about what to expect and options, they are more likely to be more open about their needs which will aid their treatment going forward and improve their experience with the service
7. Acknowledge where mistakes or missteps might have taken place	Missteps and mistakes break trust within services, so having these acknowledged and what steps can be taken to avoid this in the future, helps to regain confidence and bolster engagement
8. Ask about the potential inclusion of partners and family members during the women’s care journeys	Including and working with the women’s network can improve engagement; in supporting them to attend appointments, in encouraging and helping them to voice and be honest about their needs. As women may prefer to have loved ones involved at different points of their journey, be sure to ask throughout their time under the service if they feel this would be beneficial
9. Always consider the needs of the baby, along with the needs of the women	Working to understand women’s specific anxieties regarding parenting and the needs of the baby can increase engagement, which in turn will improve outcomes and reduce risk
10. Always keep in mind that this is a vulnerable period of time	Be sure to ask women what they feel they need or would like. When women feel able to make choices and decision making is collaborative, this improves their relationship with services and engagement
**When women aren’t engaging, be aware of how the above elements can increase their confidence in you as a practitioner and the service**	

### Strengths and limitations

This is one of the largest and most comprehensive qualitative studies of perinatal treatment engagement and outcomes. We sampled a range of CPMHTs across England and a broad range of women with different mental health problems. By taking a realist approach, this study extended previous literature in this domain by examining causal factors underlying women’s engagement with perinatal mental health services, examining what worked for whom and when.

Although the sample here is based on women who were referred to the service and opted to attend at least an initial appointment, we did have accounts from women who initially engaged well, but then disengaged, and women who didn’t initially engage well with the service but returned at a later date. These women’s accounts helped us to distinguish between what worked and what didn’t work for women once they started receiving services from CPMHTs.

Our sample was socioeconomically and ethnically representative of the range of women seen in the CPMHTs we drew from. However, like most mental health services, CPMHTs disproportionally treat White women. To fully understand the needs of ethnically diverse populations, additional research using different recruitment approaches is needed.

## Conclusion

In this study four key pillars for engagement emerged: perinatal competence, relationship building, accurate reassurance, and reliability. Consistent with Chorpita’s guidelines [32, 33] on evidence-based approaches for addressing problems in engagement, these findings suggest that key staff behaviours applied at the right time can support continued engagement and potentially contribute to better treatment outcomes. Prior to the investment in perinatal mental health services in England, women’s engagement with mental health services was low with fewer than 8 to 30% of eligible women receiving support. Getting the right, perinatally skilled, care matters, and recent results from national linked data show the CPMHTs improve women’s access to timely mental health treatment (ESMI-II unpublished results). Encouragingly, many women in this study finally found safe, trustworthy and non-stigmatising care in CPMHTs, pointing to its perinatal specific nature as a key deciding factor in their decision to engage. As mental health problems increase, disproportionately affecting vulnerable populations, it is critical to continue to ensure support is not only available, but appropriately meets the needs of those individuals.

### Electronic supplementary material

Below is the link to the electronic supplementary material.

Supplementary Material 1

## Data Availability

This study collected detailed qualitative data on a sensitive topic which included personally-identifiable information, in a limited number of identified sites. For this reason, it is not possible to anonymise the data. Therefore, the qualitative data from this study are not publicly available, but are available from the corresponding author on reasonable request.

## Abbreviations

CPMHTs: Community Perinatal Mental Health Teams
CMOCs: Context-Mechanism-Outcome Configurations
EUPD: Emotionally Unstable Personality Disorder
OCD: Obsessive Compulsive Disorder
PMH: Perinatal Mental Health
PTSD: Post Traumatic Stress Disorder
SMI: Severe Mental Illness
