# Supplementary material for: Women’s engagement with community perinatal mental health services: a realist evaluation
Source: BMC Psychiatry. 2024 Jul 8;24:492. doi: 10.1186/s12888-024-05804-1 (PMC11232178; doi:10.1186/s12888-024-05804-1)
Supplement: Supplementary file 1 — Supplementary Material 1 [file 12888_2024_5804_MOESM1_ESM.pptx]

## Slide 1
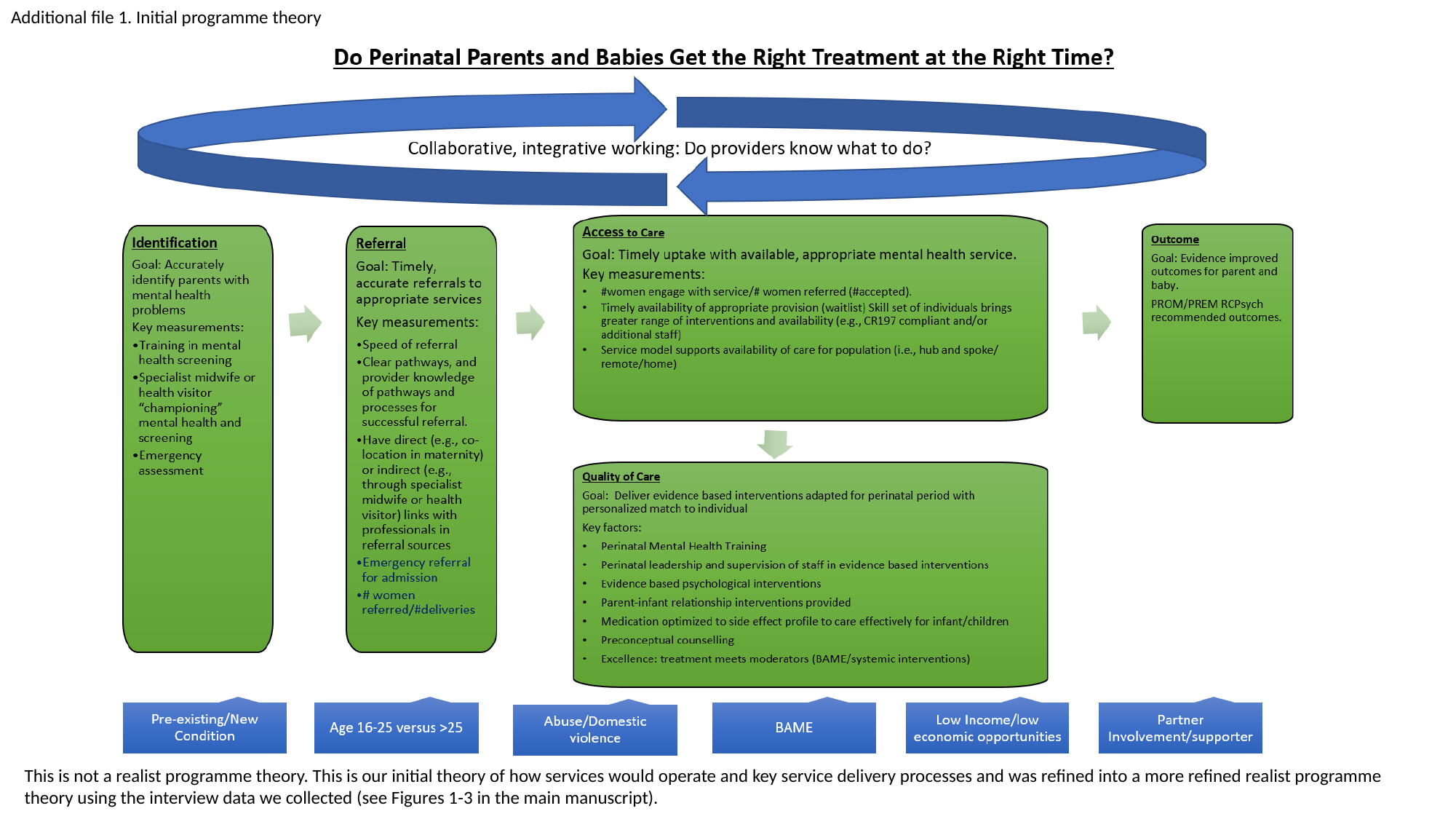

Additional file 1. Initial programme theory
This is not a realist programme theory. This is our initial theory of how services would operate and key service delivery processes and was refined into a more refined realist programme theory using the interview data we collected (see Figures 1-3 in the main manuscript).
